# Physiological evidence that three known mutations in the para-sodium channel gene confer cypermethrin knockdown resistance in *Rhipicephalus microplus*

**DOI:** 10.1186/s13071-020-04227-7

**Published:** 2020-07-22

**Authors:** Raquel Cossío-Bayúgar, Estefan Miranda-Miranda, Francisco Martínez-Ibañez, Verónica Narváez-Padilla, Enrique Reynaud

**Affiliations:** 1 Centro Nacional de Investigaciones Disciplinarias en Salud Animal e Inocuidad, Instituto Nacional de Investigaciones Forestales Agrícolas y Pecuarias (INIFAP), Cuernavaca, Morelos Mexico; 2 Servicio Nacional de Sanidad, Inocuidad y Calidad, Secretaria de Agricultura y Desarrollo Rural (SADER), México city, Ciudad de México México; 3 Centro de Investigación en Dinámica Celular, Universidad Autónoma del Estado de Morelos, Cuernavaca, Morelos México; 4 Departamento de Genética del Desarrollo y Fisiología Molecular, Instituto de Biotecnología, Universidad Nacional Autónoma de México, Cuernavaca, Morelos México

**Keywords:** *Rhipicephalus microplus*, Pyrethroid resistance, Ovary contraction, Para-sodium channel, *kdr*

## Abstract

**Background:**

Acaricide resistance is a central problem for the control of the cattle tick *Rhipicephalus microplus*. The physiological effects and phenotypes of the mutations that cause acaricide resistance are not always well understood or characterized. Single nucleotide polymorphisms (SNPs) that confer cypermethrin knockdown resistance (*kdr*) have been reported in *R. microplus*. These SNPs have been associated and correlated with pyrethroid resistance although there is no direct physiological evidence that their presence does confer *kdr* in this organism.

**Methods:**

Resistant and susceptible strain resistance profiles were obtained using the larval packet discriminating dose assay. The relevant genomic regions of the para-sodium channel were amplified using standard PCR; SNPs were detected by sequencing the corresponding amplicons. Ovary response to cypermethrin exposure/treatment was evaluated using videometrical analysis.

**Results:**

We found that the pyrethroid resistance trait is stable in a resistant reference strain after years without selection, suggesting that the resistance conferring mutations are fixed in the population. In this strain, a change in the structure of the pre-synaptic para-sodium channel caused by the G184C, the C190A and the T2134A SNPs appears to confer resistance. These mutations are absent in the susceptible strain used as control. We demonstrate that cypermethrin blocks ovary contraction in cypermethrin-susceptible ticks. We also show that ovaries from organisms that carry the *kdr* associated SNPs still contract at cypermethrin concentrations that completely block ovary contraction in the susceptible strain. The configuration of the experimental system excludes a xenobiotic detoxification mechanism.

**Conclusions:**

This is the first report that presents physiological evidence that the presence of the G184C, the C190A, and the T2134A mutations in the para-sodium channel correlates with maintaining muscle contractility in *R. microplus* exposed to cypermethrin. These SNPs may confer cypermethrin resistance in this organism by avoiding presynaptic blockage, inhibiting the flaccid muscle paralysis characteristic of this acaricide. The videometric assay that we previously validated can be used to detect more rapidly than other assays that involve larval mortality *kdr*-like cypermethrin resistant tick strains, permitting to directly assay adult pre-engorged females after they are collected on the field without waiting until eggs are laid and larvae eclose. 
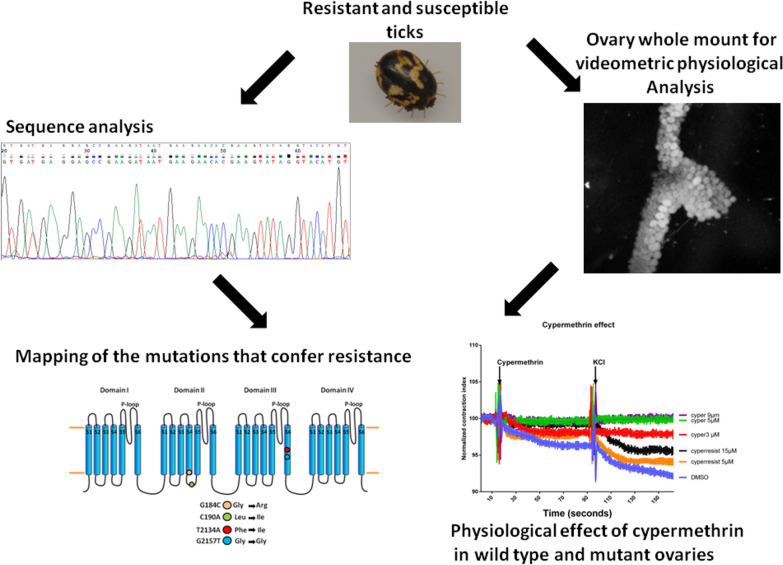

## Background

The cattle tick, *Rhipicephalus microplus*, is a bovine blood sucking parasite endemic to all tropical and subtropical livestock grazing areas around the world. Tick-infestations represent enormous economic losses to the cattle industry and considerable efforts are invested on the chemical control of this ectoparasite by the use of highly toxic pesticides, being the main strategy for tick control for decades and resulting in the selection of pesticide resistant strains. Cypermethrin (cyano-(3-phenoxyphenyl)methyl]3-(2,2-dichloroethenyl)-2,2-dimethylcyclopropane-1-carboxylate) is a type II α-cyano substituent pyrethroid used for the control of *R. microplus*. Type II pyrethroids are toxins that bind to the arthropod para-type-sodium channel inducing flaccid paralysis [1]. Cypermethrin disrupts nerve function by inhibiting channel deactivation, and stabilizing its open configuration, thus prolonging the opening of the channel and axon depolarization, rapidly inducing conduction block and failure of neuromuscular transmission causing flaccid paralysis [2–10]. Several single nucleotide polymorphisms (SNPs) in the gene that codes for the para-sodium channel that result in the substitution of single amino acids in its protein sequence have been reported to confer pyrethroid resistance in many arthropods. Homologous mutations in *R. microplus* also correlate with pyrethroid resistance but in this organism, no measurable physiological effect that correlates with the presence of these mutations and resistance has been reported. The arthropod para-sodium channel is structured into four homologous repeat domains (I–IV), each having six α-helical transmembrane segments (S1–S6) [11–13]. The reported SNPs that cause amino acid substitutions and correlate with resistance are T170C, G184C, C190A, C190G in the II domain; there is also one characterized SNP (T2134A) that is in the III domain. The G184C and the T2134A mutations occur only in resistant populations and are always genetically linked [13–17].

In this study we analyzed an acaricide resistant reference tick strain that was originally isolated from heavily infested cattle with documented records of an intensive pyrethroid control background. These ticks were toxicologically characterized at the SENASICA-SADER, the specialized laboratories for livestock pest control of the Mexican government, for susceptibility and/or resistance using the probit analysis of the larval packet discriminating dose (DD) assay [18]. We used a whole-mount preparation of the contractile tick ovary that has previously been used to assess the effect of adrenergic ligands that block oviposition and tick ovary contraction [19, 20]. This assay allows to videometrically quantitate tick ovary response to cypermethrin or any other substance. We found that resistant tick ovaries contract at cypermethrin concentrations that totally inhibit ovary contraction in susceptible strains.

## Methods

### Tick strains

An acaricide susceptible (Su) *R. microplus* tick strain, previously reported [21, 22] and a multiple acaricide-resistant tick strain dubbed San Alfonso (SA) [23] were used in this study. This strain has been maintained by continuous infestation of restrained bovines under controlled conditions without exposure to pyrethroids for the last 17 years and designated for records as the San Alfonso (SA) strain for the name of the ranch where it was originally isolated; notably, in spite of the absence of pyrethroid pressure, the ticks are still exhibiting a pyrethroid resistance phenotype according to the larval package bioassays. The SA strain is used as reference for the tick acaricide resistance monitoring programs of the Mexican Federal Government. The SA strain was reared and maintained at Departamento de Ectoparásitos y Dípteros del Servicio Nacional de Sanidad, Inocuidad y Calidad Agroalimentaria (SENASICA-SADER) and the susceptible strain was maintained at Centro Nacional de Investigación Disciplinaria en Salud Animal e Inocuidad (CENID-SAI-INIFAP). Two one-year-old *Bos taurus* (Holstein Friesian) bovines were used for tick infestations. Animal care and use was according to the mexican norm NOM-062-ZOO-1999, and its technical specifications for production, care and use of laboratory animals can be found at http://www.fmvz.unam.mx/fmvz/principal/archivos/062ZOO.PDF.

Each reference strain was obtained by infesting a bovine with 1 × 10^4^ 15-day-old larvae, engorged tick females were collected 19 to 20 days after infestation, maintained in an incubator with 80% relative humidity at 28 °C. Females are deposited on a plastic petri dish and allowed to lay embryos, that were then collected and aliquoted into 2 mg aliquots, embryos were maintained in the same incubation conditions until eclosion. Eclosed larvae are used to infest a new bovine.

### Ovary dissection

Engorged female ticks were collected 19–20 days after infestation, during this period they grow and mature over a stabulated bovine at ambient temperature and natural illumination, feeding themselves with blood to grow and mature. After 20 days of development, they become adults whose ovaries that are full of immature eggs; ticks in this condition are considered pre-engorged, in order to have enough protein to allow their eggs to mature they begin to consume copious amounts of blood and become “engorged”. We previously found [20] that the ovaries of the pre-engorged females are the most responsive to physiological treatments.

Ovaries were dissected as described by Cossio et al. [20]. Briefly, pre-engorged female ticks were washed with distilled water prior to dissection. A transversal cut between the first and second leg pairs separating the whole anterior area was made. Organs, were extruded into Jan & Jan solution (NaCl 128 mM, KCl 2 mM, 4 mM MgCl_2_, sucrose 36 mM, HEPES 5 mM pH 7.3) without Ca^2+^ to avoid neurotransmitter depletion [24], ovaries were separated from the rest of the organs and washed in Jan & Jan solutions without Ca^2+^, to be further processed.

### DNA extraction

Ten dissected ovaries were frozen with liquid nitrogen in a ceramic mortar and finely grounded. The resulting frozen powder was resuspended in *c.*500 µl in 0.1 M sodium citrate, pH 8.5, 50 mM EDTA, 0.1% SDS and 1 mg/ml PCR grade proteinase K (Thermo Fisher, Waltham, Massachusetts, USA). The tissue suspension was incubated for 2 h at 60 °C, extracted 3 times with 50% phenol pH 7.0, 50% chloroform; and then extracted once with 100% chloroform. The resulting extract was supplemented with a 10th of its volume with sodium acetate 5 M pH 5 and precipitated with 3 times its volume of absolute ethanol. The sample was centrifuged for 5 min at 12,000×*g*, washed once with 70% ethanol, air dried and resuspended in 100 µl of molecular biology grade distilled water.

### PCR amplification and SNPs determination

Oligonucleotides for the para-sodium channel relevant domains were synthesized accordingly to those reported in [13]. PCR was performed using a hot start procedure as follows: 5 min at 95 °C before adding *Taq* DNA polymerase (Thermo Fisher Scientific) using the following amplification conditions: 30 s denaturation at 95 °C, 30 s annealing at 59 °C and 30 s polymerization at 72 °C for 35 cycles, and a final extension cycle at 72 °C for 7 min.

The oligonucleotide sequences used were the following: RmNaDomainIIF1 (5’-TAC GTG TGT TCA AGC TAG CCA A-3’); RmNaDomainIIR1 (5’-ACT TTC TTC GTA GTT CTT GCC AA-3’); RmNaDomainIIIF1 (5’-AAG AGG ACC AAC CGG AAT ACG-3’); and RmNaDomainIIIR1 (5’-TCT TCT TTT GTT CAT TGA AAT TGT-3’).

PCR products were sequenced at the “Unidad de Síntesis y secuenciación de ADN del Instituto de Bitecnología” using the same oligonucleotides used for amplification.

All mutations reported in Table 1 and the rest of this work follow the naming convention for *R. microplus* based on nucleotide position within the mRNA sequence (GenBank: AF134216.2). Twenty individuals of the susceptible and the resistant population, were sampled, the corresponding amplicons were sequenced bi-directionally and analyzed for the presence of SNPs using GenBank: AF134216.2 as a reference. We found no evidence of heterozygosity.

**Table 1:** Evolution of the San Alfonso strain resistance (percentage of larval mortality) over the years without selection pressure

Year	Organophosphorous			Pyrethroids			Amidines
	Coumaphos	Diazinon	Chlorpiriphos	Cypermethrin	Deltamethrin	Flumethrin	Amitraz
2001^a^	98.60	58.6	99.70	37.50	30.80	14.50	25.20
2006^a^	20.00	0	0	0	0	0	0
2012	100	90.58	99.10	0	0	0	0
2013	91.88	91.56	99.57	0	0	0	0.62
2016	73.24	100	100	0	0	0	10.76

^a^Data from 2001 and 2006 has already been reported in [23, 37]

### Acaricide discriminant dose bioassays (DD)

Ticks were assayed and its toxicological profile was verified by acaricide discriminant dose bioassays [18]. Bioassays were performed with acaricides diluted in trichloro ethylene/olive oil (2:1) at the following pesticide concentration: coumaphos 0.2%, diazinon 0.08%, chlorpiriphos 0.2%, cypermethrin 0.05%, deltamethrin 0.09% and flumethrin 0.01%. 670 µl of each dilution was applied evenly to a 7.7 × 8.5 cm piece of filter paper; these concentrations were experimentally determined using susceptible populations as the lowest concentrations that would kill 100% of the larvae in the packet when the SENASICA-SADER program was established. Since then the concentrations have been fixed for methodological reasons. The trichloroethylene was evaporated from the filter paper in an extraction hood for 2 h. The treated papers were then folded in half and sealed on the sides with clips, forming a packet into which 100 larvae were placed; the packet was then sealed with another clip. Packets were kept at 28 °C ( ± 2.0 °C) 80–90% relative humidity for 24 h, live (motile), immobile larvae were considered dead, both populations were counted and the data was reported as the percentage of mortality for each tick group each acaricide concentration.

### Whole-mount preparation of contractile ovaries

Ovaries were dissected as described above in Jan & Jan solution (NaCl 128 mM, KCl 2 mM, 4 mM MgCl_2_, sucrose 36 mM, HEPES 5 mM pH 7.3) without Ca^2+^ to avoid neurotransmitter depletion during ovary dissection and preparation. Ovaries were loosely immobilized on a Sylgard plate in a perfusion chamber using stainless steel pins (Austerliz insect pins, minutiens 0.1mm; Fine Science tools, Vancouver, Canada); organs that may be involved in xenobiotic detoxification such as the fat body, the Malpighian tubules and the intestine were discarded. Mounted ovaries which were in Jan & Jan Ca^2+^ free solution were perfused with complete Jan & Jan solution (NaCl 128 mM, KCl 2 mM, 4 mM MgCl_2_, Sucrose 36 mM, 2 mM Ca^2+^, HEPES 5 mM pH 7.3).

### Videometric analysis of ovary response to cypermethrin

Ovary muscle contraction was videometrically recorded exactly as reported in [20]. For each ovary, the ovary area average of the first 15 frames before cypermethrin addition was used as A_0_. Normalized ovary contraction index (NCI) is defined as the ovary area value of each frame A_f_ normalized with A_0_ using the following formula: Normalized contraction index = A_f_/A_0_ × 100. The addition of Ca^2+^ increased in ovary muscle tone reflecting tissue integrity and defining ovary initial area (A_0_). NCI is defined as the area of the ovary at any moment divided by A_0_ and multiplied by 100. An NCI smaller than 100 means that the tissue has an increase in tone or contraction. An NCI bigger than 100 means that the tissue relaxes or loses tone. The effect of cypermethrin tested on muscle tone (A_1_) was measured for 70 s of exposure to treatment and averaged. Muscle contraction was induced by depolarization with 15 mM KCl. Final or maximal ovary contraction (A_2_) is defined as the average of the last 70 s after the addition of KCl. The samples were exposed to cypermethrin by perfusion with Jan & Jan solution supplemented with the corresponding cypermethrin concentrations tested (3, 5, 9 and 15 µM). Cypermethrin was dissolved in DMSO and its final concentration was 0.05% in all experiments. In all cases *n* = 9 contractile ovaries.

### Time series statistical analysis

For each data point (frame) the normalized contraction index was averaged between samples of the same treatment (*n* = 9) and its standard deviation was calculated. Control time series were obtained with susceptible contractile ovaries in (0.05% DMSO in complete Jan & Jan solution without cypermethrin). Experimental time series were compared to controls using ANOVA followed by Dunnett’s multiple comparison test accordingly to Shumway & Stoffer [25]. *P*-values ≤ 0.01 were considered significant.

## Results

### History of the toxicological profile of the San Alfonso multi-resistant strain

The resistant reference tick strain used in this work was first reported in 2002 by the “Centro Nacional de Servicios de Constatación en Salud Animal” (CONASAG-SAGARPA now SENASICA-SADER, México). This strain was toxicologically profiled using the probit analysis of the larval packet discriminating dose (DD) assay and was thereafter named San Alfonso multiresistant reference strain. At the time, this strain was resistant to the following: organophosphorous acaricides (coumaphos, diazinon and chlorpiriphos); and pyrethroids (cypermethrin, deltamethrin and flumethrin). The San Alfonso strain was also resistant to the amidine amitraz. The resistance indexes to pyrethroids of the San Alfonso strain were so high that they could not be estimated because mortality did not occur at the discriminating doses [23]. The strain was maintained and kept until present in SENASICA-SADER without exposure to acaricides. This strain has been tested periodically and its toxicological profile was determined with DD (acaricides are diluted in trichloro ethylene at the following concentrations: coumaphos 0.2%; diazinon 0.08%; chlorpiriphos 0.2%; cypermethrin 0.05%; deltamethrin 0.09%; and flumethrin 0.01%). The San Alfonso multi-resistant reference strain has been maintained for at least 15 years without selection, during this period it is still resistant to amitraz but has become susceptible again to organophosphorous acaricides (Table 1). However, it remains as resistant as it was when isolated to pyrethroids, suggesting that a mutation or mutations that confer resistance to pyrethroids have been fixed in the population. Independent sequencing of twenty individuals showed no evidence of heterozygosity.

### Cypermethrin completely inhibits ovary contraction in the susceptible strain but cannot block it in the resistant strain

Ovary contraction has been used as an assay to evaluate molecules that may inhibit oviposition in ticks [20].

**Fig. 1:**
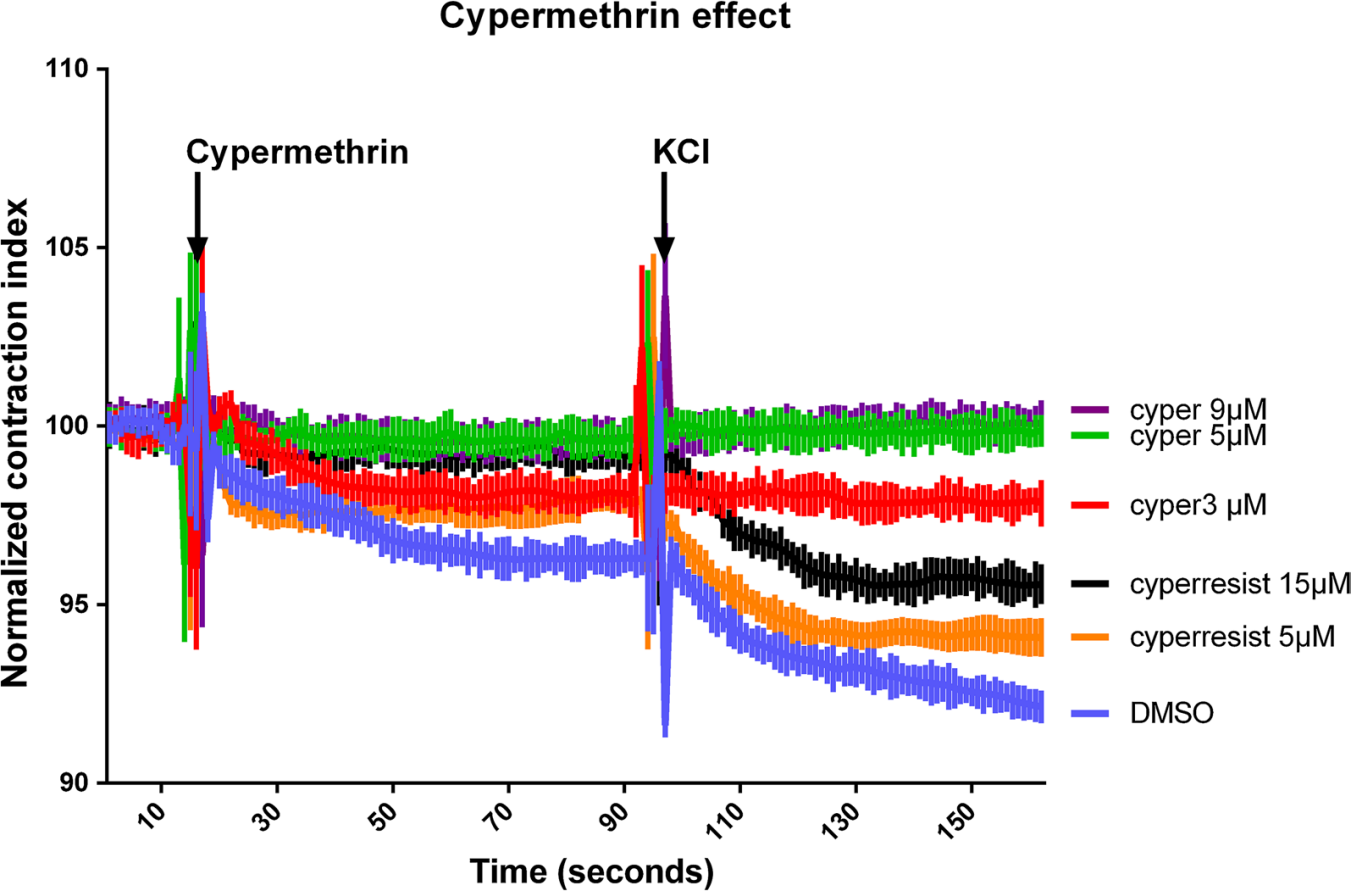
Differential sensitivity of the acaricide susceptible (Su) strain and the multiple acaricide-resistant strain (San Alfonso). Tick ovary contraction was measured using videometrical analysis. Three µM of cypermethrin is sufficient to block most of the ovary contraction induced by nerve depolarization with 15 mM KCl. Higher concentrations of 5 and 9 µM totally block ovary contraction on the susceptible strain (cyper). Ovaries from the resistant strain (cyperresist) still respond and contract at higher concentrations (5 and 15 µM). All traces (time series) represent the normalized averaged response of nine preparations for each condition. Error bars show the standard error for each video frame. All experimental conditions tested were compared to the DMSO control and all of them were significantly different from it (*P* ≤ 0.001). Abbreviations: cyper, susceptible; cyperresist, resistant; DMSO, control

Taking advantage of this technique, we evaluated the effect of several concentrations of cypermethrin on the ovary of the susceptible strain. Our results show that 3 µM of cypermethrin is sufficient to block most of the ovary contraction induced by nerve depolarization with 15 mM KCl (Fig. 1). Higher cypermethrin concentrations (5 and 9 µM) make the ovary completely unresponsive. On the other hand, ovaries from the resistant strain still responds and contract at a concentration that it is 3 times higher than the lowest observed concentration that totally inhibits ovary contraction in the susceptible strain (15 µM) (Fig. 1). Even though the SA strain is clearly resistant, cypermethrin does have an effect since all the concentrations tested had a significant reduction in their contractile index when compared with the control (*F*_(5, 7776)_ = 1115, *P* < 0.0001).

### The San Alfonso multi-resistant reference strain has mutations associated with pyrethroid resistance in the para-sodium channel

**Fig. 2:**
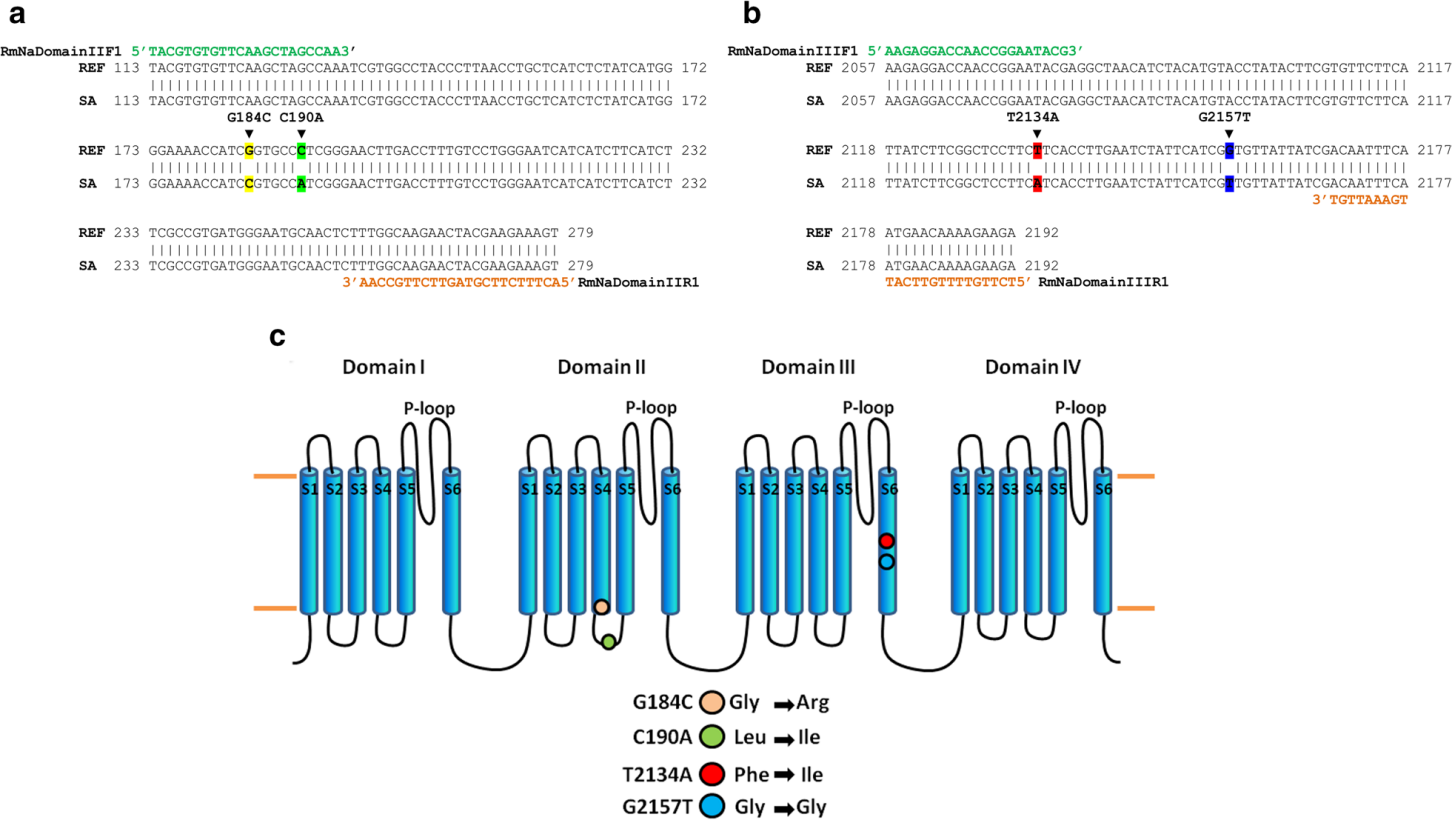
Localization of the mutations found in the San Alfonso resistant strain in the voltage-gated para-sodium channel. **a** Sequence alignment of the sequence that codes for domain II of the reference sequence (ref; GenBank: AF134216.2) and the one obtained from the San Alfonso strain (SA), the G184C nucleotide substitution is highlighted in yellow and the C190A substitution is highlighted in green. **b** Sequence alignment of the sequence that codes for domain III of the reference sequence (ref) and the one obtained from the San Alfonso strain (SA), the T2134A nucleotide substitution is highlighted in red and the G2157T substitution is highlighted in blue. **c** Transmembrane topology of the para-sodium channel: the characteristic four trans-membrane domains (I–IV) are shown as clusters of 6 blue cylinders; domain linkers are shown as black loops. Mutations are represented by colored circles

To explain the resistant phenotype of the San Alfonso multi-resistant reference strain, we decided to sequence the regions of the para-sodium channel gene that have been reported to have amino acid substitutive single nucleotide polymorphisms (SNPs) associated with pyrethroid resistance. Table 2 shows a summary of what it is known about the different SNPs that have been reported to correlate with pyrethroid resistance, and reports if there is a standardized PCR assay to detect them; it also allows us to integrate our results about the SNPs that we found present in the SA strain with previously reported homologous SNPs have been found in other pyrethroid resistant strains. All mutations reported in Table 1 and the rest of the work follow the naming convention for *R. microplus* based on nucleotide position within the mRNA sequence (GenBank: AF134216.2). We found that the resistant strain had the following amino acid substitutive mutations: G184C (Gly→Arg) and C190A (Leu→Ile) in the II domain and one mutation in the III domain: T2134A (Phe→Ile). It has been reported that the G184C (Gly→Arg) and the T2134A (Phe→Ile) always occur together and only in resistant populations [13]. In addition, we found a new silent SNP in G2157T that codes for a native Gly (Fig. 2).

**Table 2:** Mutations associated with *kdr* in ticks and insects

Domain	Nucleotide position	Nucleotide substitution	Amino acid substitution	Identified by	Standardized PCR assay	Organism and additional information (cited in)
II Susceptible population	148	C→T	Leu→Phe	[13]	None	*R. microplus*
II (super-kdr)	170	T→C	Met→Thr	[13]	[13]	Originally identified in *Musca domestica. R. microplus* [28–30]
II	184	G→C	Gly→Arg	[13]	None	*R. microplus* (Present study; [31–33]). This SNP occurs only in resistant poplulations that carry SNP T2134A
II Segment S4–5 linker	190	C→A	Leu→Ile	[16]	*R. microplus* [16]	*R. microplus* (Present study, [13, 34])
II	190	C→G	Leu→Val	[13]	None	*R. microplus* (Present study)
II Segment S4–5 linker	215	G→T	Gly→Val	[35]	[12]	*R. microplus*
III	2130	C→T	Silent	[13]	None	*R. microplus*
III Segment S6	2134	T→A	Phe→Ile	[14]	[31, 36]	*R. microplus* (Present study, [31–33]). This SNP occurs only in resistant populations that carry SNP G184C [13]
III	2157	G→T	Gly→Gly (Silent)		None	*R. microplus* (Present study)

## Discussion

The emergence of acaricide tick resistant populations is a major problem for cattle economic exploitation. These resistances permit a tick population explosion and the transmission of babesiosis and anaplasmosis reducing quality and production yields while incrementing operational costs of the livestock industry. Most resistances appear in the population within years of the first exposure to a particular acaricide and then becomes widely distributed rendering the acaricides useless. In México, pyrethroids were approved as acaricides in 1986 and seven years later (1993) the first reports of pyrethroid resistance were described using larval package dose discriminant assays, by this time most of the populations screened were already resistant to organophosphate acaricides [18, 23]. The San Alfonso multi-resistant strain was isolated during a government survey for tick eradication at a ranch located in the Mexican state of Tabasco in 2001, the survey highlighted the constant exposure of this particular isolate of ticks, for 20 years to organophosphates, 12 years to pyrethroids and 7 for amitraz; not surprisingly, the strain was resistant at the time to the three compound families. This strain has been reared and maintained since 2001 without exposure to any acaricide to be used as a multi resistant reference strain, in the CENAPA-SENASICA-SADER facilities in Jiutepec, in the state of Morelos. The strain has been evaluated periodically and resistance to organophosphates and amitraz had reverted spontaneously to be partially sensitive to these two acaricide families but remains completely resistant to pyrethroids (Table 1), The historical data suggests that when the SA strain was isolated, resistance traits to organophosphorous and amitraz were heterozygous and that resistance was conferred by the presence of alleles that were not completely fixed in the population; however, molecular changes in genes that may confer resistances to organophosphorous acaricides and amitraz have not been tracked at the molecular level and there are no preserved historical specimens. On the other hand, the stability of the pyrethroid resistance phenotype suggests that it is caused by a mutation or mutations that were fixed early in the establishment of this population and has now became homozygous. To confirm this hypothesis, we sequenced the regions of the para-sodium channel that have been associated with resistances in other tick populations [13–16]; therefore, we genotyped the extant population that it is maintained by CENAPA-SENASICA-SADER. We found that in the San Alfonso strain there are three SNPs that substitute three amino acids in the protein sequence of this channel and that these mutations were consistent with previous reports that show that these substitutions confer resistance in a similar manner of kdr mutations in other organisms. We also found a new silent nucleotide substitution that does not causes an amino acid substitution that appears to be characteristic of this strain. To gather physiological evidence that these mutations do confer resistance, we performed ovary contraction videometrical analysis, as it has been reported that this assay allows to identify molecules that block oviposition by affecting smooth muscle contraction of the ovaries.

The tick ovary is an attractive tissue for developing physiological bioassays because they account for over 50% of the mass of the pre-engorged female, they can easily be dissected and separated from other tissues such as the fat body, the Malpighian tubules and the intestines, where most xenobiotic detoxification occurs; the ovaries are enveloped in a layer of contractile smooth muscle and therefore they are an excitable tissue whose contractile response can be observed, recorded and quantified. This assay is particularly well suited to study molecules that have a pre-synaptic effect on the muscle such as adrenergics and pyrethroids. It was demonstrated in the house fly, and then in other arthropods, that cypermethrin disrupts nerve function by inhibiting the deactivation of the para-sodium channel, stabilizing its open configuration and thus prolonging the time the channel is opened, promoting a strong axon depolarization, leading to the rapid induction of axon conduction block and failure of neuromuscular transmission which in turn induces flaccid paralysis [1, 3, 7, 8, 20, 26, 27]. We found that cypermethrin also induces flaccid paralysis of the smooth muscle of the tick ovary, severely impairing ovary contraction of the susceptible strain at 3 µM and completely inhibiting all ovary reaction at 5 and 9 µM, however, ovaries of the resistant strain were still able to contract even at a 15 µM concentration that is three times more than the lowest concentration that totally inhibits ovary contraction in the susceptible strain. Type II pyrethroid resistance has been described as a knock down resistant phenotype (kdr). Type II pyrethroids prevent the closure of the para-sodium channel blocking synaptic transmission causing flaccid paralysis. Knock down resistant phenotype mutants reduce the affinity for the pyrethroid binding site of the channel reducing its effect (Table 1). The SNPs associated with kdr populations have previously been reported and correlated with resistant populations, however, to our knowledge this is the first report that correlates the presence of these mutations with a measurable physiological effect in tick tissue. This demonstrates that the toxic mechanism of type II pyrethroids in ticks and that the evolution of this type of resistance are analogous to what has been reported in other arthropods.

## Conclusions

This is the first report that presents physiological evidence that the presence of the G184C, C190A, and T2134A mutations in the para-sodium channel correlates with the ability of maintaining muscle contractility in *R. microplus* when exposed to cypermethrin. These results support the hypothesis that these SNPs confer cypermethrin resistance in this organism by permitting the deactivation of the para-sodium channel thus avoiding pre-synaptic blockage that causes flaccid muscle paralysis. This work also demonstrates that the videometric assay that we previously validated can be used to detect more rapidly *kdr*-like cypermethrin resistant tick strains than other assays that involve larval mortality, because adult pre-engorged females can be directly assayed after they are collected on the field without waiting until eggs are laid and the larvae eclose.

## Data Availability

Data supporting the conclusions of this article are included within the article. Data and materials are available upon reasonable request to the corresponding author.

## Abbreviations

SNPs: single nucleotide polymorphisms
*kdr*: cypermethrin knockdown resistance
cyano-3-phenoxyphenyl)methyl]3-(2,2-dichloroethenyl)-2,2-dimethylcyclopropane-1-carboxylate: cypermethrin
DD: larval packet discriminating dose assay
Su: acaricide susceptible
SA: San Alfonso
SENASICA-SADER: Departamento de Ectoparásitos y Dípteros del Servicio Nacional de Sanidad, Inocuidad y Calidad Agroalimentaria
CENID-SAI-INIFAP: Centro Nacional de Investigación Disciplinaria en Salud Animal e Inocuidad
NCI: normalized ovary contraction index
